# ENCAP: Computational prediction of tumor T cell antigens with ensemble classifiers and diverse sequence features

**DOI:** 10.1371/journal.pone.0307176

**Published:** 2024-07-18

**Authors:** Jen-Chieh Yu, Kuan Ni, Ching-Tai Chen

**Affiliations:** 1 Department of Bioinformatics and Medical Engineering, Asia University, Taichung, Taiwan; 2 Graduate Institute of Genomics and Bioinformatics, National Chung Hsing University, Taichung, Taiwan; 3 Center for Precision Health Research, Asia University, Taichung, Taiwan; Abdul Wali Khan University Mardan, PAKISTAN

## Abstract

Cancer immunotherapy enhances the body’s natural immune system to combat cancer, offering the advantage of lowered side effects compared to traditional treatments because of its high selectivity and efficacy. Utilizing computational methods to identify tumor T cell antigens (TTCAs) is valuable in unraveling the biological mechanisms and enhancing the effectiveness of immunotherapy. In this study, we present ENCAP, a predictor for TTCA based on ensemble classifiers and diverse sequence features. Sequences were encoded as a feature vector of 4349 entries based on 57 different feature types, followed by feature engineering and hyperparameter optimization for machine learning models, respectively. The selected feature subsets of ENCAP are primarily composed of physicochemical properties, with several features specifically related to hydrophobicity and amphiphilicity. Two publicly available datasets were used for performance evaluation. ENCAP yields an AUC (Area Under the ROC Curve) of 0.768 and an MCC (Matthew’s Correlation Coefficient) of 0.522 on the first independent test set. On the second test set, it achieves an AUC of 0.960 and an MCC of 0.789. Performance evaluations show that ENCAP generates 4.8% and 13.5% improvements in MCC over the state-of-the-art methods on two popular TTCA datasets, respectively. For the third test dataset of 71 experimentally validated TTCAs from the literature, ENCAP yields prediction accuracy of 0.873, achieving improvements ranging from 12% to 25.7% compared to three state-of-the-art methods. In general, the prediction accuracy is higher for sequences of fewer hydrophobic residues, and more hydrophilic and charged residues. The source code of ENCAP is freely available at https://github.com/YnnJ456/ENCAP.

## Introduction

Cancer is a major public health challenge that is becoming increasingly prevalent. It is the leading cause of death in many countries and is a major barrier to increasing life expectancy [1]. According to GLOBOCAN (Global Cancer Statistics) 2020, there were 19.3 million new cancer cases diagnosed in 2020 and almost 10.0 million cancer deaths [2]. GLOBOCAN predicts that the number of cancer cases will increase to 28.4 million in 2040. Modern cancer therapies such as chemotherapy, radiation therapy, hormonal therapy, and targeted therapy are lethal to tumor cells, but they can destroy normal cells in the body and produce harmful reactions such as nausea, hair loss, and fatigue [3–6]. In addition, cancer cells can develop resistance to these therapies over time, making it difficult to treat the cancer.

Targeted immunotherapy is one of the promising treatment options because of its high selectivity, efficacy, and reduced side effects [6]. T cells have the ability to identify and eliminate tumor antigens, which are presented by major histocompatibility complex (MHC) class I and class II molecules situated on the surface of antigen-presenting cells [7]. Previous studies show that peptides derived from tumor-associated antigens can induce a peptide-specific T cell immune response which destroys cancer cells [8–10]. Therefore, accurate identification of T cell antigens is an important task in developing highly efficient cancer peptide vaccines [11].

Although several *in vivo* and *in vitro* assays have been developed for the characterization of tumor T cell antigens (TTCAs), they demand a significant investment of resources, time, and labor. On the other hand, computational methods are attracting increasing attention due to their great convenience and high efficiency [12–15]. TTAgP 1.0 uses a random forest (RF) model with relatively simple features including amino acid composition (AAC) and several physicochemical features derived from peptides for the identification of TTCAs [16]. iTTCA-Hybrid uses support vector machines (SVM) and RF with five types of features including AAC, dipeptide composition, pseudo amino acid composition, distribution of amino acid properties in sequences, and physicochemical properties derived from the AAindex database [17] for TTCA prediction [18]. iTTCA-RF [19] considers more features, such as global protein sequence descriptors, grouped amino acid composition, grouped dipeptide composition, grouped tripeptide composition, and pseudo-amino acid composition. These features are then processed by MRMD (Maximum-Relevance-Maximum-Distance) [20] to determine an optimal feature subset of 263 numeric values for training and independent test based on RF. TAP 1.0 [21] applies information gain [22] on 544 features from AAindex, resulting in a reduced subset of 10 properties for training and testing based on 15 machine learning (ML) algorithms, within which quadratic discriminant analysis outperforms the rest. iTTCA-MFF [23] uses 50 physicochemical properties and pairwise energy content matrix for encoding, and LASSO (Least Absolute Shrinkage and Selection Operator) algorithm to select the optimal feature subset. The selected features are provided to an SVM model to identify TTCA.

Though existing studies have achieved a certain degree of success, some of them have the drawbacks of limited selection of feature types and a lack of a systematic approach for the selection of feature subset and optimization of ML models. More importantly, there is still potential for improvement in the prediction accuracy. In this study, we present ENCAP (ENsemble predictors for tumor T Cell Antigen Prediction), a TTCA predictor constructed with ensemble classifiers on diverse sequence-based features. A total of 57 different feature types were considered for feature engineering, producing a vector of 4349 numeric values for each sequence. Next, feature ranking was performed and the feature subset for the follow-up ML process was determined with a heuristic feature subset selection algorithm. Six different ensemble classifiers such as random forest and extreme gradient boosting were applied in this study. The hyperparameters of ML models were then optimized with a Bayesian optimization algorithm based on the selected feature subset. Noteworthily, our ML models have been applied on two different TTCA datasets which consist of TTCAs obtained from TANTIGEN [24, 25], but have different criteria in the acquisition of non-TTCAs. For the first dataset, our predictor achieves a Matthew’s correlation coefficient (MCC) of 0.522 for independent test, producing a 4.8% improvement compared to the state-of-art method, iTTCA-RF. For the second dataset, our predictor achieves an MCC of 0.789 for independent test, yielding a 13.5% improvement over the state-of-art method, PSRTTCA. For the third independent test dataset of 71 experimentally validated TTCAs collected from literature, ENCAP achieves accuracy up to 0.873, producing improvements in between 12% and 25.7% over three state-of-the-art methods. Furthermore, the feature analysis indicates that the feature subset of ENCAP is primarily composed of physicochemical properties, with several features specifically related to hydrophobicity and amphiphilicity. It is demonstrated that the differences in the criteria for non-TTCAs between the two datasets can lead to differences in the selected feature subset, especially the compositional features. This situation also highlights the effectiveness of ENCAP in performing adaptive feature selection for the target dataset.

## Materials and methods

### Datasets

In this study, we employed two publicly available datasets to develop and assess ENCAP. The first dataset [18], containing 592 TTCAs and 393 non-TTCAs, was randomly partitioned into a cross validation (CV) dataset and an independent test set using an 80% and 20% split. The CV dataset, termed DS1-CV, consisted of 470 TTCAs and 318 non-TTCAs; the independent test set, termed DS1-IND, consisted of 122 TTCAs and 75 non-TTCAs. The second dataset consisted of two subsets: DS2-CV, comprising 474 TTCAs and 474 non-TTCAs for cross validation, and DS2-IND, comprising an additional 118 TTCAs and 118 non-TTCAs for independent test [26]. The peptide length distributions of TTCAs and non-TTCAs for DS1 and DS2 are illustrated in S1 and S2 Figs, respectively. It can be observed the lengths for a majority of the TTCAs and non-TTCAs fall within a range of 8 to 10 residues.

For both DS1 and DS2, the TTCAs were obtained from TANTIGEN (Tumor T-cell Antigen Database), and the non-TTCAs were obtained from Immune Epitope Database (IEDB) [27, 28]. The two datasets primarily differ in the acquisition of non-TTCAs. Non-TTCAs from DS1 were obtained with a single criterion: T cell antigens with no known association with any disease. In contrast, non-TTCAs from DS2 were collected using multiple criteria, such as antigens not associated with terms such as ‘tumor’, ‘cancer’, ‘carcinoma’, and ‘metastasis’, validation through *in vitro* and *in vivo* assays, presentation by MHC-I (Major Histocompatibility Complex-I) molecules, having *Homo sapiens* as antigen source and host, and a length of 9 to 14 amino acids. Given that DS1 and DS2 have been employed in multiple studies, we assessed our models using both datasets.

Apart from the two datasets, an additional test dataset of 73 experimentally validated TTCAs collected from literature were obtained from a previous study [26]. Two of the sequences were removed because they consisted of non-standard amino acid, resulting in 71 TTCAs termed DS3-IND. This dataset served as an additional test set for benchmark analysis of prediction models trained with DS1-CV and DS2-CV as well as existing predictors.

### Overview of the method

**Fig 1:**
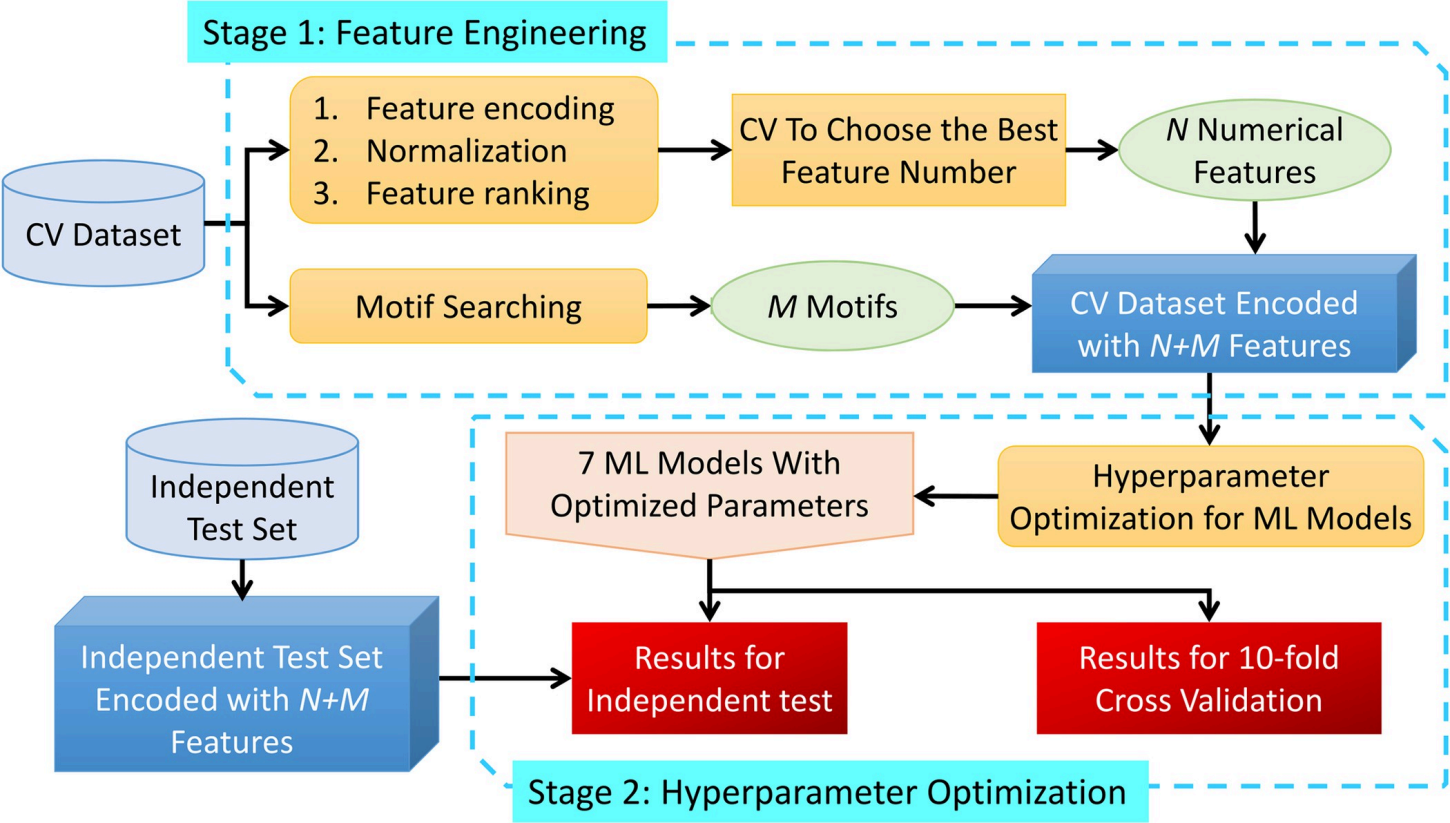
Flowchart of ENCAP.

ENCAP utilizes a two-stage process to construct the prediction model, as illustrated in Fig 1. The first stage is feature engineering and the second stage is hyperparameter optimization for ML models. In the first stage, sequences from the CV dataset were converted to various compositional and physicochemical features, followed by data normalization and feature ranking. Next, the best feature number was determined with cross validation using 7 ML models. The *N* selected features were combined with *M* features based on motifs, which were obtained with a motif searching algorithm. The *N+M* features were deemed the best feature subset, which were then used for hyperparameter optimization of ML models in stage 2. A total of 6 ensemble prediction models were considered, including CatBoost (CB) [29], gradient boosting classifier (GBC) [30], extra trees (ET) [31], extreme gradient boosting (XGB) [32], light gradient boosting machine (LGBM) [33], random forest (RF) [34]. Apart from the above, a statistical method, linear discriminant analysis (LDA) [35], was also used for comparison. The hyperparameters of 7 ML models were optimized with a Bayesian optimization algorithm on the CV dataset using the selected feature subset from the first stage. After the hyperparameters for each ML model were determined, 10-fold CV and independent test were performed and the results were benchmarked with various evaluation measures. The pseudocode of ENCAP is shown in S1 Text.

### Feature encoding

Converting amino acid sequences into numerical representations is crucial in accurate prediction of TTCA using machine learning algorithms. Various compositional and physicochemical features were used to encode peptides, including peptide length, amino acid composition (AAC), di-peptide composition (DPC), composition of k-spaced amino acid pairs (CKSAAP) [36], amphiphilic pseudo amino acid composition (APAAC) [37–39], composition of k-spaced amino acid group pairs (CKSAAGP) [40, 41], Composition-Transition-Distribution (CTD) [42], conjoint triad (CTriad) [43], dipeptide deviation from expected mean (DDE) [44], grouped amino acid composition (GAAC) [45], grouped di-peptide composition (GDPC) [46], distance distribution of repeats (DDR) [47], residue repeats index (RRI) [47], Shannon entropy of a residue (SER) [47], Shannon entropy of a property (SEP) [47], quasi-sequence order (QSO) [48], instability index [49], Boman index [50, 51], isoelectric point [52], and aromaticity. These features were obtained with iFeature [53], pFeature [47], and modlAMP [54] packages. The entire list of 57 feature types and the size of each of them (number of numeric values) are listed in S1 Table. A feature vector of 4349 entries was generated for each sequence.

Using a diverse set of feature types for sequence encoding is advantageous because different feature types can capture distinct aspects of the peptide sequences, such as composition, physicochemical properties, and structural patterns. By combining multiple feature types, a more comprehensive representation of the sequences is generated, which can mitigate biases introduced by individual encoding methods and potentially improve the generalization ability of ML models.

Apart from the above features, a binary vector associated with sequence motifs was generated. Sequence motifs of DS1 and DS2 were obtained with STREME [55], a motif searching tool which finds ungapped motifs that are enriched in the dataset. STREME was applied to positive and negative samples separately to obtain respective list of motifs ranging from 3 to 20 amino acids. After combining the two motif lists, a binary vector was generated in which the presence and absence of a motif in a given sequence were represented by 1 and 0, respectively. The binary vector was also taken as input for machine learning models.

### Normalization

Robust normalization was applied to compositional and physicochemical features with *RobustScaler* from *Scikit-learn* package [56] of Python. RobustScaler converts a feature value *x*_*i*_ to *y*_*i*_ using the following equation.

$$y_{i}=\frac{x_{i}-Median(X)}{Q3(X)-Q1(X)},$$ (1)

in which *Q3*(*X*) and *Q1*(*X*) stand for the 3rd quartile and the 1st quartile of feature *X*. The scaled values have a median of zero and an interquartile range of one across all sequences.

### Machine learning methods

Ensemble predictors were used in this study because of their accuracy, robustness towards noises, and good model generalizability. We used 6 ensemble predictors in this study, including RF, ET, GBC, LGBM, XGB, and CB. In addition, a statistical model, LDA, was also employed for comparison. RF comprises an extensive ensemble of decision trees, and its prediction is obtained by calculating the average of the outcomes produced by individual trees. ET is similar to RF except for two notable distinctions: 1) every tree is trained using the entire training dataset rather than a bootstrap sample, and 2) the top-down node splitting is determined through a random process, as opposed to relying on a scoring function like information gain or Gini impurity. GBC, LGBM, XGB, and CB are different implementations of boosting algorithms that combines several weak learners into strong learners, in which each new model is trained to minimize the loss function such as mean squared error or cross-entropy of the previous model using gradient descent. LDA attempts to find a linear combination of features that best separates classes in a dataset while minimizing the variance within each class. Implementation of these models was based on *Scikit-learn* package of Python.

### Heuristic feature subset selection

The Boruta package [57] implements a wrapper approach utilizing a random forest algorithm for feature ranking. The algorithm iteratively eliminates features that are statistically less relevant than randomized features, resulting in a ranked feature list based on feature importance. The algorithm with its default parameters was applied to analyze features (except for the motifs) for DS1-CV and DS2-CV, yielding two ranked feature lists.

Next, a heuristic algorithm was applied to determine the best feature numbers of DS1-CV and DS2-CV for machine learning. For a given dataset, the ranked features were expressed as an ordered set *F* = {*f*_*1*_, *f*_*2*_, … *f*_*4335*_}, sorted in descending order according to feature importance. The feature subset of the top *N* features is defined as *S*_*N*_ = {*f*_*1*_, *f*_*2*_, …*f*_*N*_} where *f*_*i*_ ∈ *F*. Iterative five-fold cross validation runs using *S*_*N*_ was performed based on the six ensemble predictors, i.e., RF, ET, GBC, LGBM, XGB, and CB. The default hyperparameters of the Scikit-learn package were used for all the models. The cross validation experiments were carried out with PyCaret [58] and the results were evaluated with MCC. Let *MCC*_*N*_^*i*^ be the MCC for ML model *i* using *S*_*N*_, The best MCC based on *S*_*N*_ was defined as

$${Best\_MCC}_{N=}\underset{i}{\max}\left({MCC}_{N}^{i}\right)$$ (2)

Where ML model *i* corresponds to any of the six ML models (RF, ET, GBC, LGBM, XGB, and CB). The process was repeated iteratively, with *N* ranging from 50 to 410 in increments of 20. The best feature subset (BFS) was defined as

$$BFS=S_{j},where\;{Best\_MCC}_{j}=\underset{k}{\max}{(Best\_MCC}_{k})$$ (3)

As a result, two feature subsets were generated by applying the heuristic algorithm to DS1-CV and DS2-CV. The pseudocode of the algorithm is shown in S2 Text. The purpose of feature subset selection is to determine the best feature number for each dataset and avoid the lengthy process of machine learning model optimization. This is the reason why we used the default hyperparameters of the six machine learning models for the task. Moreover, using ML models of different rationale has the advantage that the selected feature subsets are not biased towards a particular model.

### Hyperparameter optimization and independent test

Once the feature subset was determined, hyperparameters of ML models were optimized with Optuna [59], an automatic hyperparameter optimization software package for sampling search space and pruning unpromising trials. We used the tree-structured Parzen estimator (TPE) algorithm [60], a Bayesian optimization method, as the search algorithm of Optuna because it is more likely to reach global optimum than randomized search, and is more time-efficient than grid search. The default parameters of TPE were used during the process. After the hyperparameters were determined for each model, ten-fold cross validation was performed to evaluate each predictor.

To perform independent test, an additional training process using the optimized parameters of each model on the entire DS1-CV or DS2-CV was performed. The resulting models, with the advantage of using 10% more training data compared to the models obtained from cross validation, were used to conduct independent test on DS1-IND and DS2-IND.

### Performance evaluation

For benchmark comparison, prediction results were evaluated with accuracy, precision, recall or sensitivity, F1-score, and MCC (Matthew’s Correlation Coefficient) defined as follows:

$$Accuracy=\frac{\mathrm{TP}+\mathrm{TN}}{\mathrm{TP}+\mathrm{TN}+\mathrm{FP}+\mathrm{FN}}$$ (4)

$$Precision=\frac{\mathrm{TP}}{\mathrm{TP}+\mathrm{FP}}$$ (5)

$$Recall=Sensitivity=\frac{\mathrm{TP}}{\mathrm{TP}+\mathrm{FN}}$$ (6)

$$Specificity=\frac{\mathrm{TN}}{\mathrm{TN}+\mathrm{FP}}$$ (7)

$$F1‐score=\frac{2\times \mathrm{Precision}\times \mathrm{Recall}}{\mathrm{Precision}+\mathrm{Recall}}$$ (8)

$$MCC=\frac{\mathrm{TP}\times \mathrm{TN}-\mathrm{FP}\times \mathrm{FN}}{\sqrt{\left(\mathrm{TP}+\mathrm{FP})(\mathrm{TP}+\mathrm{FN})(\mathrm{TN}+\mathrm{FP})(\mathrm{TN}+\mathrm{FN}\right)}}$$ (9)

where TP, TN, FP, and FN stand for the numbers of true positives, true negatives, false positives, and false negatives, respectively. Accuracy, precision, recall, specificity, and F1 range from 0 to 1, where a larger value indicates better predictive capability. MCC ranges from -1 to 1, representing completely negative and completely positive correlations, respectively, and an MCC of 0 represents random correlation. In addition, AUC (Area Under the receiver operating characteristic Curve) [61], a non-parametric and threshold independent measure, was also included for evaluation. An AUC value ranging from 0.5 to 1 is indicative of a model with good prediction performance.

## Results

### Selected features and motifs

Cross validation results of DS1-CV and DS2-CV using different feature numbers were evaluated with MCC, and the results are shown in S3 Fig. The analysis reveals that selecting 150 features for DS1-CV and 210 features for DS2-CV results in the highest MCC, thereby constituting the selected feature subset. Using the selected feature subset, the best MCCs achieved by the 7 machine learning models are 0.535 and 0.757 for DS1-CV and DS2-CV, respectively.

STREME generated 9 motifs for TTCAs from DS1-CV, including FATP, AEEA, ALQP, AVI, LMK, SLADTN, VFGI, RLAE, LLD, and 2 motifs for non-TTCAs from DS1-CV, including IPRHL and ALAG. For DS2-CV, STREME yielded 3 motifs from TTCAs, including LMK, ADV, and AGIGIL, and 5 motifs from non-TTCAs, including AQID, EFP, AVADE, VDEV, and ITD. Interestingly, the motifs from both datasets are quite different because the non-TTCA sequences were collected based on different criteria, resulting in dissimilar sequence patterns for the negatives. The sizes of feature vectors for DS1-CV and DS2-CV are 161 (150+11) and 218 (210+8), respectively.

### Performance evaluation for cross validation

The hyperparameters of 7 ML models trained with DS1-CV are listed in S2 Table. Evaluation results of cross validation on DS1-CV for all the ML models are shown in Table 1. It can be observed ET yields accuracy, precision, F1-score, and MCC of 0.760, 0.751, 0.821, and 0.488, respectively, all of which are the highest among all the methods. CB and GBC have AUCs of 0.793 and 0.803, respectively, comparable to ET but their MCCs are 3.3% and 3.5% lower than ET. XGB has the highest recall but suffers from the lowest precision among all the methods.

**Table 1:** Evaluation results of cross validation on DS1-CV for all the ML models. Bald face indicates the highest value among all the methods.

Model	Accuracy	Precision	Recall	F1-score	Specificity	AUC	MCC
ET	**0.760**	**0.751**	0.906	**0.821**	0.557	0.795	**0.488**
CB	0.746	0.736	0.909	0.813	0.519	0.793	0.455
GBC	0.746	0.745	0.888	0.810	0.550	**0.803**	0.453
XGB	0.732	0.708	**0.949**	0.811	0.421	0.788	0.433
LGBM	0.732	0.738	0.870	0.799	0.544	0.775	0.422
RF	0.719	0.739	0.830	0.782	**0.566**	0.714	0.397
LDA	0.664	0.715	0.743	0.729	0.563	0.683	0.288

The hyperparameters of 7 ML models trained with DS2-CV are listed in S3 Table. Evaluation results of cross validation on DS2-CV for all the ML models are shown in Table 2. ET yields accuracy, precision, F1-score, and MCC of 0.852, 0.856, 0.852, and 0.708, respectively, all of which are again the highest among all the methods. GBC, RF, CB, and XGB show marginally lower MCC and AUC (within 1% difference), indicating that these ensemble machine learning models are somewhat comparable to ET in cross validation. By comparing the evaluation results of DS1-CV with DS2-CV, it is obvious the specificities for all the models on DS1-CV are below 0.6, significantly lower than those obtained on DS2-CV, which exceed 0.82. Moreover, all the models produce ~10% lower precision for DS1 compared to DS2. The above observations indicate a higher propensity for false positive predictions on DS1, a characteristic specific to the dataset.

**Table 2:** Evaluation results of cross validation on DS2-CV for all the ML models. Bald face indicates the highest value among all the methods.

Model	Accuracy	Precision	Recall	F1-score	Specificity	AUC	MCC
ET	**0.852**	**0.856**	0.848	**0.852**	0.857	0.928	**0.708**
GBC	0.847	0.852	0.846	0.849	0.853	0.927	0.698
RF	0.847	0.843	**0.860**	0.851	**0.862**	0.923	0.698
CB	0.847	0.851	0.842	0.846	0.839	**0.930**	0.697
XGB	0.839	0.848	0.828	0.838	0.824	0.914	0.681
LGBM	0.836	0.838	0.833	0.835	0.833	0.909	0.675
LDA	0.821	0.826	0.814	0.820	0.812	0.901	0.644

### Performance evaluation for independent test

Benchmark results for independent test on DS1-IND with 7 ML models are shown in Table 3. Results for iTTCA-Hybrid, TTAgP 1.0, and LDA are also listed in the table for comparison because they were developed and benchmarked on the same dataset. It can be seen the best performing method, GBC, generates the highest MCC of 0.522 and the highest accuracy of 0.766. CB and ET yield slightly lower MCCs of 0.508 and 0.488, respectively. We further analyze the correlation between the prediction confidence (or probability output) for each model and the true positive rate, calculated by the number of actual TTCA divided by the total number of sequences predicted within the range of the prediction confidence. It can be observed from S4A Fig. that the prediction confidence for each model in general has positive correlation to the true positive rate. LDA has the highest true positive rate for the MCC range of 0 to 0.2 than all the other methods. The situation is unfavorable and explains why LDA produces the lowest MCC for independent test. GBC, CB, and ET outperform the state-of-art method, iTTCA-RF, in MCC by 4.8%, 3.4%, and 1.4%, respectively. iTTCA-RF has the highest precision and specificity among all the methods, and yet it generates a recall of 0.836, considerably lower than the recall for GBC, CB, and ET which range from 0.975 to 1.000. This is the major reason why GBC, CB, and ET outperform iTTCA-RF in terms of MCC.

**Table 3:** Evaluation results of independent test on DS1-IND for all the ML models. Bald face indicates the highest value among all the methods.

Model	Accuracy	Precision	Recall	F1-score	Specificity	AUC	MCC
GBC	**0.766**	0.729	0.992	**0.840**	0.400	0.768	**0.522**
CB	0.756	0.718	**1.000**	0.836	0.360	0.777	0.508
ET	0.756	0.726	0.975	0.832	0.400	0.757	0.488
XGB	0.751	0.721	0.975	0.829	0.387	0.744	0.477
LGBM	0.751	0.724	0.967	0.828	0.400	0.766	0.472
RF	0.746	0.717	0.975	0.826	0.373	0.735	0.465
LDA	0.695	0.704	0.877	0.781	0.400	0.656	0.320
iTTCA-RF	0.756	**0.785**	0.836	0.810	**0.627**	0.780	0.474
iTTCA-Hybrid	0.736	0.765	0.828	0.795	0.587	**0.783**	0.428
TTAgP 1.0	0.711	0.756	0.787	0.771	0.587	0.747	0.379

Table 4 shows the benchmark results for independent test on DS2-IND with 7 ML models and two other existing methods, PSRTTCA and TAP 1.0, both of which were developed and benchmarked on the same dataset. It can be seen ET is the best performing model because its MCC, AUC, accuracy, and recall are 0.789, 0.960, 0.890, and 0.966, respectively, all of which are the highest among all the methods. All 7 models present good correlations between the prediction confidence and true positive rate, as illustrated in S4B Fig. Similar to the prediction results of DS1-IND, ET, GBC, and CB are the top 3 methods judging by MCC, and they outperform state-of-art method, PSRTTCA, by 13.5%, 12.7%, and 10.5% in MCC, respectively. Moreover, the improvements of ET, GBC, and CB over PSRTTCA in AUC are 7.5%, 6.6%, and 7.2%, respectively. The precision of PSRTTCA is 0.827, comparable to the precision of ET, GBC, and CB, and yet its recall is 0.822, which is 14.4%, 14.4%, and 11.0% lower than the three methods, respectively. The situation explains why PSRTTCA has much lower MCC than the three methods. The above results on DS1-IND and DS2-IND indicate that significantly improved recall is the major reason why the proposed methods compare favorably over the state-of-art approach.

**Table 4:** Evaluation results of independent test on DS2-IND for all the ML models. Bald face indicates the highest value among all the methods.

Model	Accuracy	Precision	Recall	F1-score	Specificity	AUC	MCC
ET	**0.890**	0.838	**0.966**	**0.897**	0.814	**0.960**	**0.789**
GBC	0.886	0.832	**0.966**	0.894	0.805	0.951	0.781
CB	0.877	0.840	0.932	0.884	0.822	0.957	0.759
XGB	0.886	**0.903**	0.864	0.883	**0.907**	0.952	0.772
LGBM	0.873	0.900	0.839	0.868	**0.907**	0.944	0.748
RF	0.864	0.836	0.907	0.870	0.822	0.943	0.731
LDA	0.860	0.851	0.873	0.862	0.848	0.919	0.721
PSRTTCA	0.827	0.827	0.822	0.824	0.832	0.885	0.654
TAP 1.0	0.738	0.758	0.676	0.715	0.786	0.817	0.483

To predict sequences from DS3-IND (consisting of 71 experimentally validated TTCAs collected from literature), we employed ET and GBC trained with DS1-CV (termed DS1-ET and DS1-GBC, respectively) because the two are the best for cross validation and independent test on DS1 judging by MCC, respectively. ET trained with DS2-CV, termed DS2-ET, were also used for benchmark comparison because the model outperforms the rest for both cross validation and independent test on DS2. DS1-ET, DS1-GBC, and DS2-ET correctly predict 62, 60, and 58 TTCAs from the dataset, respectively, namely, the accuracy of the three models ranges from 0.873 to 0.817. On the other hand, PSRTTCA, iTTCA-RF, iTTCA-Hybrid, and TAP 1.0 predict 55, 52, 52, and 45 TTCAs out of 71 actual TTCAs in DS3-IND. The accuracy of the four existing methods ranges from 0.753 to 0.616, indicating that our proposed methods significantly improve existing methods in prediction accuracy for DS3-IND.

## Discussions

### Data visualization with selected features

**Fig 2:**
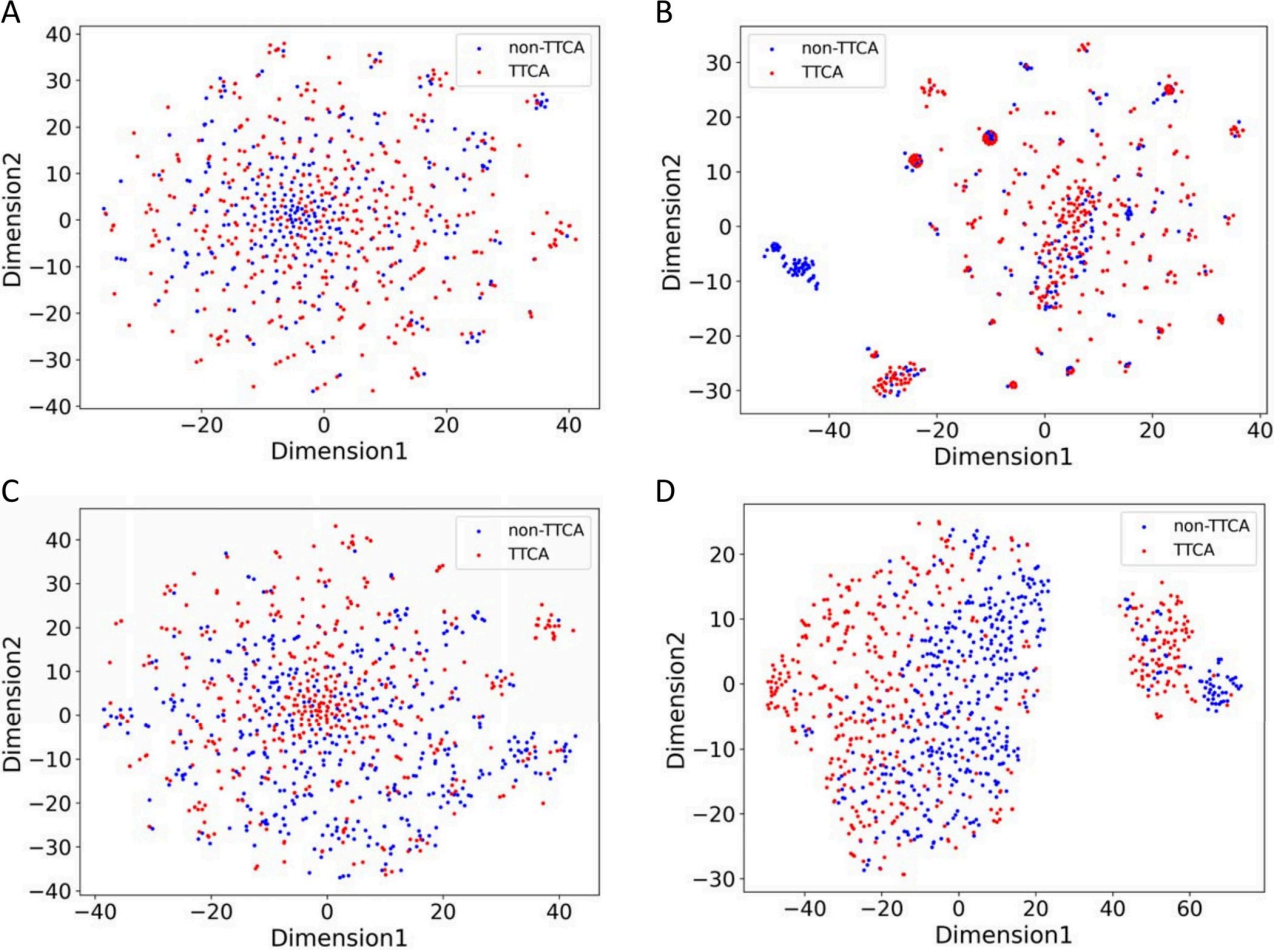
t-SNE distributions of (A) DS1-CV using 4349 features, (B) DS1-CV using 150 selected features, (C) DS2-CV using 4349 features, and (D) DS2-CV using 210 selected features.

In this study, each sequence is originally encoded with various biological, physicochemical, and compositional features, resulting in a feature vector of 4349 numeric values. The improved performances of our predictors on both DS1-IND and DS2-IND are largely attributed to the selection of numerical features. Through feature engineering, a total of 150 and 210 feature values were obtained. The t-distributed stochastic neighbor embedding (t-SNE) [62, 63] was applied to validate the efficacy of the selected feature subsets to the discrimination of TTCAs. Fig 2 illustrates the t-SNE distributions of TTCAs and non-TTCAs on 2D planes using either the complete set of 4349 features or the selected feature subsets. Within these plots, red and blue spots represent TTCAs and non-TTCAs, respectively. Notably, Fig 2B and 2D highlight the enhanced separation achieved by the selected feature subsets, facilitating clearer differentiation between the distributions of TTCAs and non-TTCAs. Conversely, when employing all 4349 features for t-SNE analysis, the distributions of TTCAs and non-TTCAs exhibit serious overlap, as shown in Fig 2A and 2C. Such results indicate the importance and efficacy of the selected feature subsets, which can benefit ML models in accurate prediction of TTCAs.

### Analysis of selected feature types

The entire lists of the 161 selected features for DS1 and the 218 selected features for DS2 are shown in S4 and S5 Tables, respectively. The former belongs to 16 different feature types and the latter belongs to 25 different feature types (excluding motifs), among which 8 feature types are in common. The top 5 most frequent feature types of DS1 and DS2 are shown in Table 5. It can be observed 4 of the top 5 most frequent feature types for DS1 and DS2 belong to the common feature types, indicating a certain degree of similarity between the two datasets. The entire lists of feature types used for machine learning on DS1 and DS2 are shown in S6 Table. These feature types are mostly physicochemical properties, for example, CTD (Composition-Transition-Distribution) [42] is constructed with properties including hydrophobicity, normalized van der Waals volume, polarity, polarizability, charge, secondary structure and solvent accessibility. Other selected features of physicochemical properties include ABHPRK (Acidic, Basic, Hydrophobic, Polar, aRomatic, Kink-inducer) [64], MSW (amino acid scale based on a principal component analysis of the Molecular Surface-based Weighted holistic invariant molecular descriptor) [65], Z5 [66], and Z3 [67]. Apart from the general physicochemical properties, hydrophobicity seems to be one of the important attributes among the selected features. For example, the Aliphatic_Index [68] and OVPC_Aliphatic (OVerlapping Property Composition) [69] for DS1 and APAAC (Amphiphilic Pseudo-Amino Acid Composition) [48] for DS2 are related to hydrophobicity.

**Table 5:** The selected numbers and sizes of features for the top 5 most frequent feature types (excluding motifs) used for machine learning on DS1 and DS2.

DS1			DS2		
Feature Type	Selected Num.	Size	Feature Type	Selected Num.	Size
DDE	91	400	CTDD*	78	195
MSW*	19	30	Ez*	22	30
Ez*^a^	9	30	Cougar*	18	30
QSO*	8	46	z5*	17	75
Cougar*	6	30	CTDC	12	39

*common feature types between DS1 and DS2

^a^potential that assesses energies of insertion of amino acid side chains into lipid bilayers

The major difference between the feature types for DS1 and DS2 is the presence of compositional features. For example, DS1 consists of 91 DDE features (Dipeptide Deviation from Expected mean) [70] while none are selected for DS2. DS2 consists of a much smaller number of compositional features, such as 5 APAAC features (Amphiphilic Pseudo-Amino Acid Composition), 2 AAC features (Amino Acid Composition), and 2 GAAC (Grouped Amino Acid Composition) [45] features. Such difference is attributed to the feature ranking algorithm, Boruta, which ranks the contribution of each feature based on its discriminating capability for a given dataset. Specifically, the differences in the criteria for non-TTCAs between DS1 and DS2 lead to different sequence patterns, thus altering the feature ranks generated by Boruta. The situation also highlights the flexibility of our approach in selecting suitable features for the target dataset.

### Feature importance analysis

**Fig 3:**
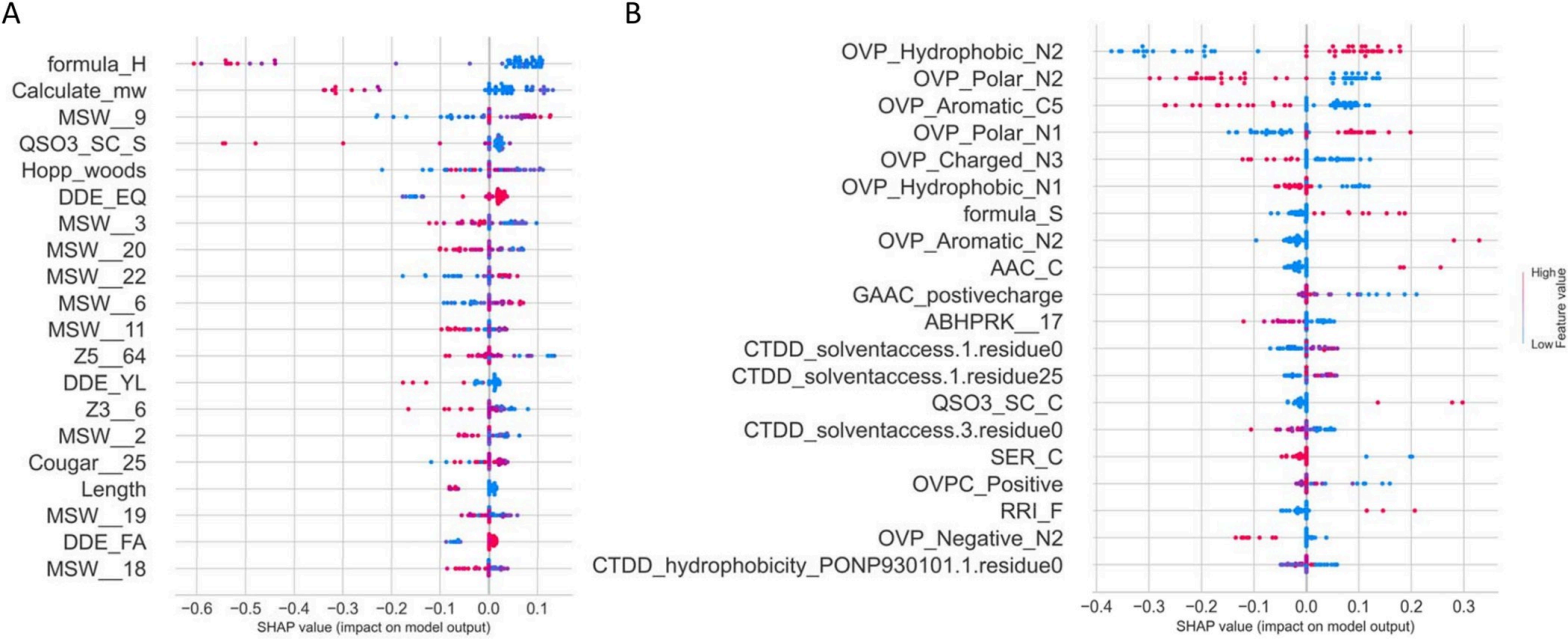
The beeswarm plots of SHAP values for the top 20 features based on A) DS1 and B) DS2.

SHAP (SHapley Additive exPlanations) [71] analysis is known as a powerful framework to provide information about how features can affect the output of the model. Positive SHAP values signify that a feature contributed to augmenting the model’s output, making a positive prediction more likely, whereas negative values indicate the feature’s role in diminishing the output, increasing the likelihood of a negative prediction. The absolute magnitude of the SHAP values quantifies the extent of each feature’s influence, namely, feature importance. Fig 3 illustrates the SHAP values for the top 20 features based on DS1 and DS2. It can be seen the top 20 features for DS1 (Fig 3A) are mostly associated with physicochemical properties such as MSW, Z3, and Z5. On the other hand, 8 of the top 20 features for DS2 (Fig 3B) are OVPs corresponding to the hydrophobic, polar, charged, and aromatic residues on the N-terminus of peptides (N1, N2, and N3). Several CTD features corresponding to solvent accessibility and hydrophobicity also have high SHAP values for DS2. The analysis reveals different feature contributions for the two datasets, reflecting different underlying patterns or characteristics that are relevant to the prediction task.

### Peptide property with respect to prediction capability

**Fig 4:**
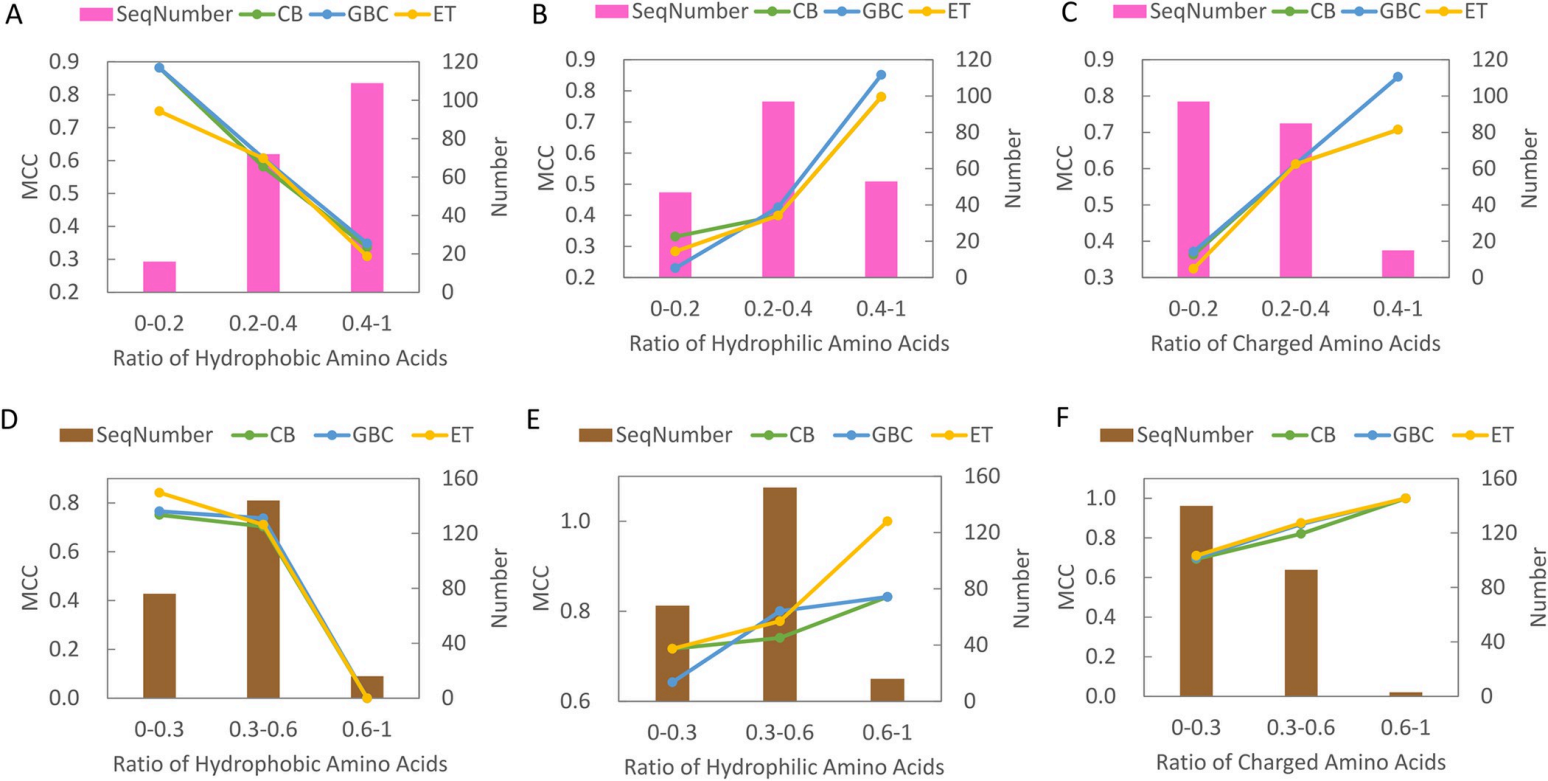
Analysis of prediction performance evaluated with MCC with respect to three different peptide properties. Panels A, B, and C corresponds to ratios of hydrophobic, hydrophilic, and charged amino acids for peptides from DS1-IND. Panels D, E, and F corresponds to ratios of hydrophobic, hydrophilic, and charged amino acids for peptides from DS2-IND.

Prediction results of the three most accurate models on independent tests, CB, GBC, and ET, were further analyzed with respect to three peptide properties. The peptide property is characterized by the ratios of hydrophobic, hydrophilic, and charged residues within a peptide. Hydrophobic amino acids include V, I, L, M, F, W, and C; hydrophilic amino acids include R, N, D, E, Q, H, K, S, and T; charged amino acids include E, D, R, K, and H. The analysis is applied to peptides from DS1-IND and DS2-IND and the results are illustrated in Fig 4. It can be observed the prediction capability for the ML models are improved for peptides of fewer hydrophobic residues, and more hydrophilic and charged residues. Moreover, the phenomena for DS1-IND (Fig 4A–4C) and DS2-IND (Fig 4D–4F) are remarkably similar. The analysis also suggests potential directions for future enhancements, particularly in refining the prediction accuracy for peptides characterized by a higher number of hydrophobic residues and a reduced presence of hydrophilic and charged residues.

## Conclusion

Cancer immunotherapy is a type of cancer treatment that uses the body’s own immune system to fight cancer cells. Accurate identification of TTCA is an important task for cancer immunotherapy because peptides derived from TTCAs can induce a peptide-specific T cell immune response which neutralizes tumor cells. In this study, we present ENCAP, a ML method built with ensemble classifiers and diverse sequence features for TTCA prediction. In the stage of feature engineering, sequences were encoded with a total of 4349 numeric values from 57 different feature types, followed by feature ranking with Boruta and a heuristic feature subset selection process. In addition, the sequence motifs from TTCAs were used to generate bit vectors as part of the features. The second stage utilized the selected feature subset for the optimization of hyperparameters for the 7 ML models (CB, GBC, ET, XGB, LGBM, RF, and LDA) using a Bayesian optimization approach. ENCAP was applied to two TTCA datasets, DS1 and DS2, from the public domain which have quite different criteria for the acquisition of non-TTCAs. For DS1-IND, the best ML model, XGB, yields 0.766, 0.729, 0.992, and 0.522 for accuracy, precision, recall, and MCC, respectively. For DS2-IND, the best ML model, ET, yields 0.890, 0.838, 0.966, and 0.789, for accuracy, precision, recall, and MCC, respectively. The improvements in MCC over the state-of-art methods for independent tests of DS1 and DS2 are 4.8% and 13.5%, respectively. The situation is largely attributed to the considerable increase in recall for the proposed models. For DS3, the dataset of 71 experimentally validated TTCAs gathered from the literature, our top-performing models exhibit accuracy ranging from 0.817 to 0.873. In contrast, existing methods yield accuracy within the range of 0.616 to 0.753. The above results suggest our models produce significant improvements over existing methods in identifying TTCAs.

The efficacy of the selected features is further validated with t-SNE plots, for which the 150 and 210 selected features from DS1 and DS2, respectively, demonstrate enhanced separation compared to original features. Our feature analysis reveals that the selected feature subset consists of mostly physicochemical properties, along with several features specifically corresponding to hydrophobicity and amphiphilicity. The feature subsets for DS1 and DS2 mainly differ in the selection of compositional features. The situation shows that different criteria for non-TTCAs can result in different sequence patterns, which can affect the discriminating capability of features. The SHAP analysis also reveals different feature contributions for the two datasets, reflecting different underlying patterns or characteristics that are relevant to the prediction task.

This study is the first to conduct separate benchmark comparisons of predictors on two TTCA datasets. Our results not only demonstrate significant improvements in prediction accuracy for three independent tests, but provide evidences for the effectiveness of ENCAP in adaptively selecting features for the target dataset. Furthermore, it is demonstrated that the prediction accuracy for peptides of a higher proportion of hydrophobic residues and a lower occurrence of hydrophilic and charged residues is limited, indicating a need for further improvement. As more experimentally validated TTCAs become available, it can be anticipated that employing advanced deep learning architectures and large language models may enhance the performance of TTCA predictions. These techniques could potentially capture complex sequence patterns and long-range dependencies that are challenging for traditional machine learning models. Ultimately, continued research at this intersection of cancer immunotherapy and machine learning could pave the way for more effective identification of TTCA candidates, accelerating the development of novel immunotherapeutic strategies against cancer.

## Supporting information

S1 Fig Peptide length distributions of TTCAs and non-TTCAs for A) DS1-CV and B) DS1-IND.
(DOCX)

S2 Fig Peptide length distributions of TTCAs and non-TTCAs for A) DS2-CV and B) DS2-IND.
(DOCX)

S3 Fig MCCs of the best machine learning model evaluated with cross validation using different feature numbers on A) DS1-CV and B) DS2-CV.
(DOCX)

S4 Fig Prediction confidence of 7 ML models on (A) DS1-IND and (B) DS2-IND.
(DOCX)

S1 Table Details of 57 feature types used in the study. (DOCX)

S2 Table Hyperparameters of ML models trained with DS1-CV. (DOCX)

S3 Table Hyperparameters of ML models trained with DS2-CV. (DOCX)

S4 Table List of 161 selected feature subset for DS1. (DOCX)

S5 Table List of 218 selected feature subset for DS2. (DOCX)

S6 Table The selected numbers and sizes of feature types from the selected feature subsets of DS1 and DS2. (DOCX)

S1 Text Pseudocode of ENCAP. (DOCX)

S2 Text Pseudocode of the feature subset selection algorithm. (DOCX)
