# Supplementary figures and images for: ENCAP: Computational prediction of tumor T cell antigens with ensemble classifiers and diverse sequence features
Source: PLoS One. 2024 Jul 18;19(7):e0307176. doi: 10.1371/journal.pone.0307176 (PMC11257298; doi:10.1371/journal.pone.0307176)

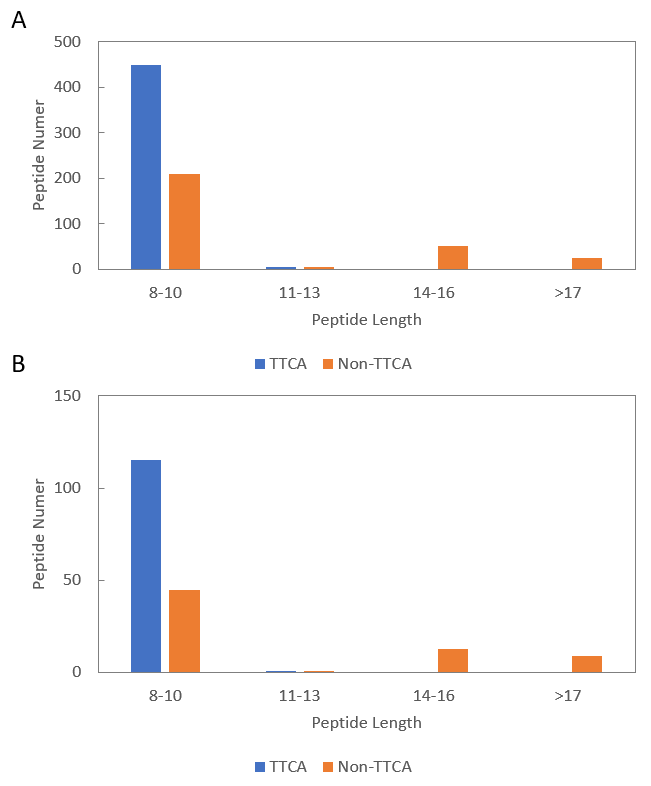


**S1 Fig.** Peptide length distributions of TTCAs and non-TTCAs for A) DS1-CV and B) DS1-IND.

Supplement: S1 Fig — Peptide length distributions of TTCAs and non-TTCAs for A) DS1-CV and B) DS1-IND. (DOCX) [file pone.0307176.s001.docx]

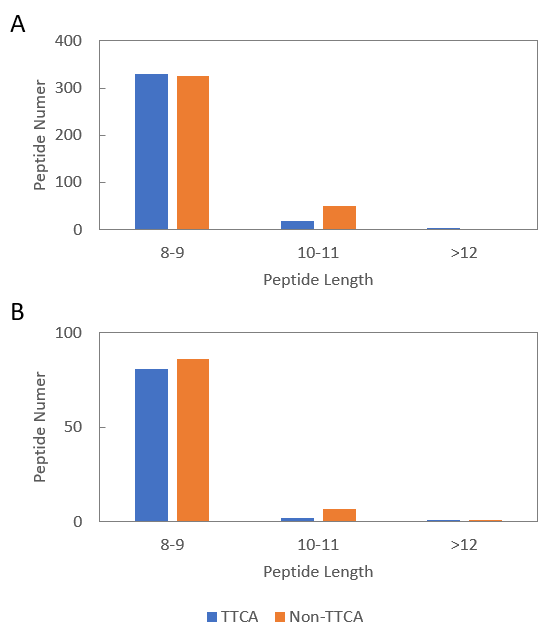


**S2 Fig.** Peptide length distributions of TTCAs and non-TTCAs for A) DS2-CV and B) DS2-IND.

Supplement: S2 Fig — Peptide length distributions of TTCAs and non-TTCAs for A) DS2-CV and B) DS2-IND. (DOCX) [file pone.0307176.s002.docx]
