# Supplementary material for: ENCAP: Computational prediction of tumor T cell antigens with ensemble classifiers and diverse sequence features
Source: PLoS One. 2024 Jul 18;19(7):e0307176. doi: 10.1371/journal.pone.0307176 (PMC11257298; doi:10.1371/journal.pone.0307176)
Supplement: S3 Fig — MCCs of the best machine learning model evaluated with cross validation using different feature numbers on A) DS1-CV and B) DS2-CV. (DOCX) [file pone.0307176.s003.docx]

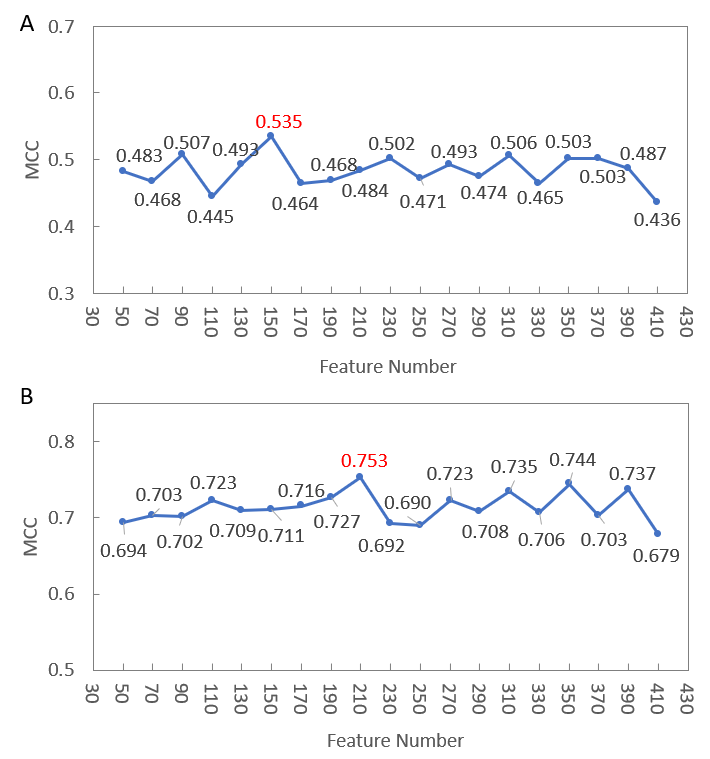


**S3 Fig.** MCCs of the best machine learning model evaluated with cross validation using different feature numbers on A) DS1-CV and B) DS2-CV. The highest MCC is shown in red.
