# Supplementary material for: ENCAP: Computational prediction of tumor T cell antigens with ensemble classifiers and diverse sequence features
Source: PLoS One. 2024 Jul 18;19(7):e0307176. doi: 10.1371/journal.pone.0307176 (PMC11257298; doi:10.1371/journal.pone.0307176)
Supplement: S4 Fig — Prediction confidence of 7 ML models on (A) DS1-IND and (B) DS2-IND. (DOCX) [file pone.0307176.s004.docx]

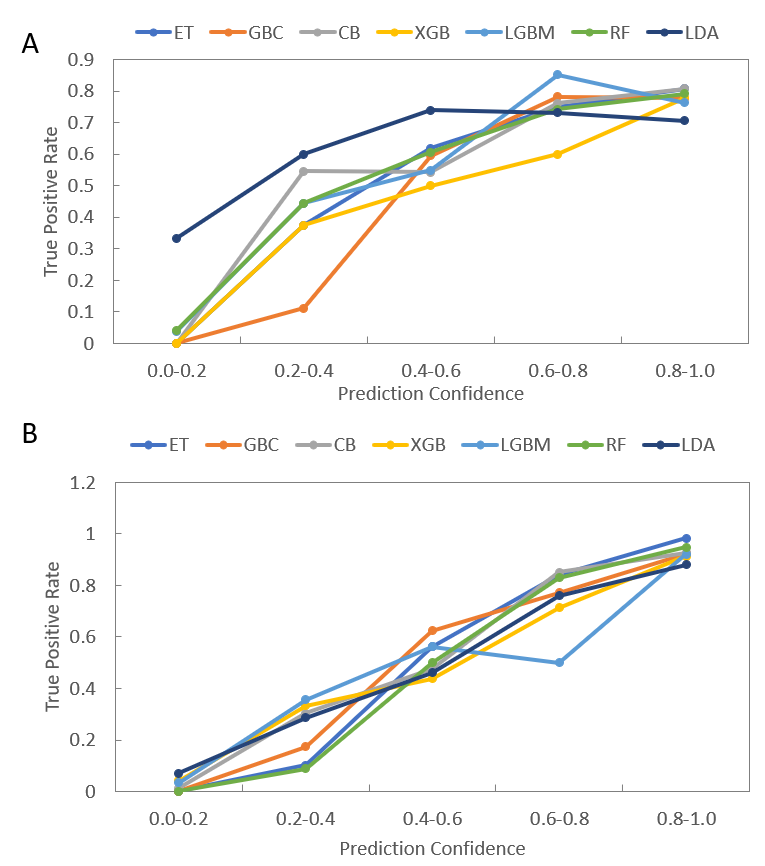


**S4 Fig.** Prediction confidence of 7 ML models on (A) DS1-IND and (B) DS2-IND. Prediction confidence is the probability output of each machine learning model. True positive rate is calculated by the number of TTCA divided by the total number of sequences predicted within the range of the prediction confidence.
