# Supplementary material for: ENCAP: Computational prediction of tumor T cell antigens with ensemble classifiers and diverse sequence features
Source: PLoS One. 2024 Jul 18;19(7):e0307176. doi: 10.1371/journal.pone.0307176 (PMC11257298; doi:10.1371/journal.pone.0307176)
Supplement: S1 Table — (DOCX) [file pone.0307176.s005.docx]

**S1 Table.** Details of 57 feature types used in the study. I, P, and M in the package column represent iFeature, pFeature, and ModelAMP, respectively. The size refers to the number of numeric values for a particular feature type.

| Feature Type | Full Name | Size | Package | Ref. |
| --- | --- | --- | --- | --- |
| CKSAAP | Composition of k-spaced Amino Acid Pairs | 1600 | I | [1] |
| DDE | Dipeptide Deviation from Expected Mean | 400 | P | [2] |
| DPC | Dipeptide Composition | 400 | P | [3] |
| CTriad | Conjoint Triad | 343 | I | [4] |
| KSCTriad | K-Spaced Conjoint Triad | 343 | I | [5] |
| CTDD | Composition/Transition/**Distribution** | 195 | I | [6] |
| CKSAAGP | Composition of k-Spaced Amino Acid Group Pairs | 150 | I | [5] |
| GTPC | Grouped Tri-Peptide Composition | 125 | I | [7] |
| ABHPRK | Acidic, Basic, Hydrophobic, Polar, aRomatic, Kink-inducer | 105 | M | [8] |
| OVP | Overlapping property | 100 | inhouse | [9] |
| Z5 | The extended five dimensional Z-scale for amino acids | 75 | M | [10] |
| QSO | Quasi-Sequence Order | 46 | P | [11] |
| CTDC | **Composition**/Transition/Distribution | 39 | I | [6] |
| CTDT | Composition/**Transition**/Distribution | 39 | I | [6] |
| Cougar | Modlabs inhouse selection of global peptide descriptors | 30 | M | [8] |
| Ez | Empirical residue-based potential | 30 | M | [12] |
| MSW | The molecular surface based WHIM descriptor | 30 | M | [13] |
| Z3 | The extended three dimensional Z-scale for amino acids | 30 | M | [14] |
| APAAC | Amphiphilic Pseudo-Amino Acid Composition | 26 | I | [15] |
| GDPC | Grouped Di-Peptide Composition | 25 | I | [7] |
| Geary | Geary correlation | 24 | I | [16] |
| Moran | Moran correlation | 24 | I | [17] |
| NMBroto | Normalized Moreau-Broto Autocorrelation | 24 | I | [17] |
| AAC | Amino Acid Composition | 20 | I | [15] |
| DDR | Distance Distribution of Residues | 20 | P | [18] |
| RRI | Repetitive Residue Information | 20 | P | [18] |
| SER | Shannon Entropy at Residue Level | 20 | P | [18] |
| SEP | Shannon Entropy at Protein Level | 20 | P | [18] |
| OVPC | Overlapping property C-terminus | 10 | inhouse | [9] |
| GAAC | Grouped Amino Acid Composition | 5 | I | [19] |
| formula | formula | 5 | M | [8] |
| Shannon-Entropy | Shannon Entropy of a Protein | 1 | P | [18] |
| Length | Length | 1 | M | [20] |
| Calculate_mw | The molecular weight [g/mol] | 1 | M | [21] |
| Calculate_charge | Charge | 1 | M | [22] |
| Isoelectric_point | Isoelectric_point | 1 | M | [20] |
| Instability_index | Instability_index | 1 | M | [23] |
| Aromaticity | Aromaticity | 1 | M | [20] |
| Aliphatic_Index | Aliphatic index | 1 | M | [24] |
| Hydrophobic | Hydrophobic | 1 | M | [20] |
| AASI | Amino acid selectivity index scale | 1 | M | [25] |
| Argos | Argos | 1 | M | [26] |
| Bulkiness | Amino acid side chain bulkiness scale | 1 | M | [27] |
| Charge_phys | Amino acid charge at pH 7. | 1 | M | [28] |
| Charge_acid | Amino acid charge at acidic pH | 1 | M | [28] |
| Flexibility | amino acid side chain flexibilitiy scale | 1 | M | [29] |
| Gravy | GRAVY hydrophobicity amino acid scale | 1 | M | [30] |
| Levitt_alpha | Levitt amino acid alpha-helix propensity scale | 1 | M | [31] |
| MSS | A graph-theoretical index that reflects topological shape and size of amino acid side chains | 1 | M | [32] |
| Polarity | Amino acid polarity scale | 1 | M | [27] |
| Refractivity | Relative amino acid refractivity values | 1 | M | [33] |
| TM_tend | Amino acid transmembrane propensity scale | 1 | M | [34] |
| Boman_Index | Boman index | 1 | M | [35] |
| Eisenberg | The Eisenberg hydrophobicity consensus amino acid scale | 1 | M | [36] |
| Hopp_woods | Hopp-Woods amino acid hydrophobicity scale | 1 | M | [37] |
| Janin | Janin hydrophobicity amino acid scale | 1 | M | [38] |
| Kytedoolittle | Kyte & Doolittle hydrophobicity amino acid scale | 1 | M | [30] |

References

1. Argos, Patrick, J. K. Mohana Rao, and Paul A. Hargrave. 1982. “Structural Prediction of Membrane-Bound Proteins.” *European Journal of Biochemistry* 128 (2–3): 565–75. https://doi.org/10.1111/j.1432-1033.1982.tb07002.x.
2. Basith, Shaherin, Gwang Lee, and Balachandran Manavalan. 2022. “STALLION: A Stacking-Based Ensemble Learning Framework for Prokaryotic Lysine Acetylation Site Prediction.” *Briefings in Bioinformatics* 23 (1): bbab376. https://doi.org/10.1093/bib/bbab376.
3. Bhaskaran, R., and P.k. Ponnuswamy. 1988. “Positional Flexibilities of Amino Acid Residues in Globular Proteins.” *International Journal of Peptide and Protein Research* 32 (4): 241–55. https://doi.org/10.1111/j.1399-3011.1988.tb01258.x.
4. Boman, H. G., D. Wade, I. A. Boman, B. Wåhlin, and R. B. Merrifield. 1989. “Antibacterial and Antimalarial Properties of Peptides That Are Cecropin-Melittin Hybrids.” *FEBS Letters* 259 (1): 103–6. https://doi.org/10.1016/0014-5793(89)81505-4.
5. Chen, Ke, Lukasz A. Kurgan, and Jishou Ruan. 2008. “Prediction of Protein Structural Class Using Novel Evolutionary Collocation-Based Sequence Representation.” *Journal of Computational Chemistry* 29 (10): 1596–1604. https://doi.org/10.1002/jcc.20918.
6. Chou, K. C. 2001. “Prediction of Protein Cellular Attributes Using Pseudo-Amino Acid Composition.” *Proteins* 43 (3): 246–55. https://doi.org/10.1002/prot.1035.
7. Chou, Kuo-Chen. 2000. “Prediction of Protein Subcellular Locations by Incorporating Quasi-Sequence-Order Effect.” *Biochemical and Biophysical Research Communications* 278 (2): 477–83. https://doi.org/10.1006/bbrc.2000.3815.
8. “Co-AMPpred for in Silico-Aided Predictions of Antimicrobial Peptides by Integrating Composition-Based Features | BMC Bioinformatics | Full Text.” n.d. Accessed June 15, 2023. https://bmcbioinformatics.biomedcentral.com/articles/10.1186/s12859-021-04305-2.
9. Cock, Peter J. A., Tiago Antao, Jeffrey T. Chang, Brad A. Chapman, Cymon J. Cox, Andrew Dalke, Iddo Friedberg, et al. 2009. “Biopython: Freely Available Python Tools for Computational Molecular Biology and Bioinformatics.” *Bioinformatics* 25 (11): 1422–23. https://doi.org/10.1093/bioinformatics/btp163.
10. Cornette, James L., Kemp B. Cease, Hanah Margalit, John L. Spouge, Jay A. Berzofsky, and Charles DeLisi. 1987. “Hydrophobicity Scales and Computational Techniques for Detecting Amphipathic Structures in Proteins.” *Journal of Molecular Biology* 195 (3): 659–85. https://doi.org/10.1016/0022-2836(87)90189-6.
11. Dean, Scott N., and Scott A. Walper. 2020. “Variational Autoencoder for Generation of Antimicrobial Peptides.” *ACS Omega* 5 (33): 20746–54. https://doi.org/10.1021/acsomega.0c00442.
12. Dubchak, I, I Muchnik, S R Holbrook, and S H Kim. 1995. “Prediction of Protein Folding Class Using Global Description of Amino Acid Sequence.” *Proceedings of the National Academy of Sciences of the United States of America* 92 (19): 8700–8704.
13. Eisenberg, David, Robert M. Weiss, Thomas C. Terwilliger, and William Wilcox. 1982. “Hydrophobic Moments and Protein Structure.” *Faraday Symposia of the Chemical Society* 17 (0): 109–20. https://doi.org/10.1039/FS9821700109.
14. Garg, Aarti, Manoj Bhasin, and Gajendra P. S. Raghava. 2005. “Support Vector Machine-Based Method for Subcellular Localization of Human Proteins Using Amino Acid Compositions, Their Order, and Similarity Search *.” *Journal of Biological Chemistry* 280 (15): 14427–32. https://doi.org/10.1074/jbc.M411789200.
15. Garg, Aarti, and Gajendra P. S. Raghava. 2008. “A Machine Learning Based Method for the Prediction of Secretory Proteins Using Amino Acid Composition, Their Order and Similarity-Search.” *In Silico Biology* 8 (2): 129–40.
16. Guruprasad, Kunchur, B.V.Bhasker Reddy, and Madhusudan W. Pandit. 1990. “Correlation between Stability of a Protein and Its Dipeptide Composition: A Novel Approach for Predicting in Vivo Stability of a Protein from Its Primary Sequence.” *Protein Engineering, Design and Selection* 4 (2): 155–61. https://doi.org/10.1093/protein/4.2.155.
17. Heather, James M., Paisley T. Myers, Feng Shi, Mohammad Ovais Aziz-Zanjani, Keira E. Mahoney, Matthew Perez, Benjamin Morin, et al. 2019. “Murine Xenograft Bioreactors for Human Immunopeptidome Discovery.” *Scientific Reports* 9 (1): 18558. https://doi.org/10.1038/s41598-019-54700-2.
18. Hellberg, Sven, Michael Sjoestroem, Bert Skagerberg, and Svante Wold. 1987. “Peptide Quantitative Structure-Activity Relationships, a Multivariate Approach.” *Journal of Medicinal Chemistry* 30 (7): 1126–35. https://doi.org/10.1021/jm00390a003.
19. Hopp, T P, and K R Woods. 1981. “Prediction of Protein Antigenic Determinants from Amino Acid Sequences.” *Proceedings of the National Academy of Sciences* 78 (6): 3824–28. https://doi.org/10.1073/pnas.78.6.3824.
20. Horne, D. S. 1988. “Prediction of Protein Helix Content from an Autocorrelation Analysis of Sequence Hydrophobicities.” *Biopolymers* 27 (3): 451–77. https://doi.org/10.1002/bip.360270308.
21. IKAI, Atsushi. 1980. “Thermostability and Aliphatic Index of Globular Proteins.” *The Journal of Biochemistry* 88 (6): 1895–98. https://doi.org/10.1093/oxfordjournals.jbchem.a133168.
22. Juretić, Davor, Damir Vukičević, Nada Ilić, Nikolinka Antcheva, and Alessandro Tossi. 2009. “Computational Design of Highly Selective Antimicrobial Peptides.” *Journal of Chemical Information and Modeling* 49 (12): 2873–82. https://doi.org/10.1021/ci900327a.
23. Kyte, Jack, and Russell F. Doolittle. 1982. “A Simple Method for Displaying the Hydropathic Character of a Protein.” *Journal of Molecular Biology* 157 (1): 105–32. https://doi.org/10.1016/0022-2836(82)90515-0.
24. Lee, Tzong-Yi, Zong-Qing Lin, Sheng-Jen Hsieh, Neil Arvin Bretaña, and Cheng-Tsung Lu. 2011. “Exploiting Maximal Dependence Decomposition to Identify Conserved Motifs from a Group of Aligned Signal Sequences.” *Bioinformatics* 27 (13): 1780–87. https://doi.org/10.1093/bioinformatics/btr291.
25. Levitt, Michael. 2002. “Conformational Preferences of Amino Acids in Globular Proteins.” Research-article. ACS Publications. American Chemical Society. World. May 1, 2002. https://doi.org/10.1021/bi00613a026.
26. Li, Fuyi, Xudong Guo, Dongxu Xiang, Miranda E. Pitt, Arnold Bainomugisa, and Lachlan J. M. Coin. 2022. “Computational Analysis and Prediction of PE_PGRS Proteins Using Machine Learning.” *Computational and Structural Biotechnology Journal* 20 (January):662–74. https://doi.org/10.1016/j.csbj.2022.01.019.
27. Manavalan, Balachandran, Shaherin Basith, Tae Hwan Shin, Leyi Wei, and Gwang Lee. 2019. “mAHTPred: A Sequence-Based Meta-Predictor for Improving the Prediction of Anti-Hypertensive Peptides Using Effective Feature Representation.” *Bioinformatics (Oxford, England)* 35 (16): 2757–65. https://doi.org/10.1093/bioinformatics/bty1047.
28. McMeekin, Thomas L., Mildred Wilensky, and Merton L. Groves. 1962. “Refractive Indices of Proteins in Relation to Amino Acid Composition and Specific Volume.” *Biochemical and Biophysical Research Communications* 7 (2): 151–56. https://doi.org/10.1016/0006-291X(62)90165-1.
29. “Predicting Protein–Protein Interactions Based Only on Sequences Information | PNAS.” n.d. Accessed June 14, 2023. https://www.pnas.org/doi/10.1073/pnas.0607879104.
30. Raychaudhury, Chandan, Asok Banerjee, Partha Bag, and Syamal Roy. 1999. “Topological Shape and Size of Peptides:  Identification of Potential Allele Specific Helper T Cell Antigenic Sites.” *Journal of Chemical Information and Computer Sciences* 39 (2): 248–54. https://doi.org/10.1021/ci980052w.
31. Sandberg, Maria, Lennart Eriksson, Jörgen Jonsson, Michael Sjöström, and Svante Wold. 1998. “New Chemical Descriptors Relevant for the Design of Biologically Active Peptides. A Multivariate Characterization of 87 Amino Acids.” *Journal of Medicinal Chemistry* 41 (14): 2481–91. https://doi.org/10.1021/jm9700575.
32. Senes, Alessandro, Deborah C. Chadi, Peter B. Law, Robin F. S. Walters, Vikas Nanda, and William F. DeGrado. 2007. “Ez, a Depth-Dependent Potential for Assessing the Energies of Insertion of Amino Acid Side-Chains into Membranes: Derivation and Applications to Determining the Orientation of Transmembrane and Interfacial Helices.” *Journal of Molecular Biology* 366 (2): 436–48. https://doi.org/10.1016/j.jmb.2006.09.020.
33. Sokal, Robert R., and Barbara A. Thomson. 2006. “Population Structure Inferred by Local Spatial Autocorrelation: An Example from an Amerindian Tribal Population.” *American Journal of Physical Anthropology* 129 (1): 121–31. https://doi.org/10.1002/ajpa.20250.
34. Timmons, Patrick Brendan, and Chandralal M. Hewage. 2021. “ENNAACT Is a Novel Tool Which Employs Neural Networks for Anticancer Activity Classification for Therapeutic Peptides.” *Biomedicine & Pharmacotherapy* 133 (January):111051. https://doi.org/10.1016/j.biopha.2020.111051.
35. Velez Rueda, Ana Julia, Franco Leonardo Bulgarelli, Nicolás Palopoli, and Gustavo Parisi. 2023. “CaviDB: A Database of Cavities and Their Features in the Structural and Conformational Space of Proteins.” *Database* 2023 (January):baad010. https://doi.org/10.1093/database/baad010.
36. Zaliani, A., and E. Gancia. 1999. “MS-WHIM Scores for Amino Acids:  A New 3D-Description for Peptide QSAR and QSPR Studies.” *Journal of Chemical Information and Computer Sciences* 39 (3): 525–33. https://doi.org/10.1021/ci980211b.
37. Zhao, Gang, and Erwin London. 2006. “An Amino Acid ‘Transmembrane Tendency’ Scale That Approaches the Theoretical Limit to Accuracy for Prediction of Transmembrane Helices: Relationship to Biological Hydrophobicity.” *Protein Science* 15 (8): 1987–2001. https://doi.org/10.1110/ps.062286306.
38. Zimmerman, J. M., Naomi Eliezer, and R. Simha. 1968. “The Characterization of Amino Acid Sequences in Proteins by Statistical Methods.” *Journal of Theoretical Biology* 21 (2): 170–201. https://doi.org/10.1016/0022-5193(68)90069-6.
