# Supplementary material for: ENCAP: Computational prediction of tumor T cell antigens with ensemble classifiers and diverse sequence features
Source: PLoS One. 2024 Jul 18;19(7):e0307176. doi: 10.1371/journal.pone.0307176 (PMC11257298; doi:10.1371/journal.pone.0307176)
Supplement: S2 Table — (DOCX) [file pone.0307176.s006.docx]

**S2 Table.** Hyperparameters of ML models trained with DS1-CV

| Model | Parameters |
| --- | --- |
| Extra Trees Classifier | bootstrap=True,  ccp_alpha=0.0,  criterion= 'entropy'  max_depth= 7,  max_features= 0.5383500573727464,  max_leaf_nodes=None,  max_samples=None,  min_impurity_decrease= 1.6651333259776753e-06,  min_impurity_split=None,  min_samples_leaf=3,  min_samples_split=2  min_weight_fraction_leaf=0.0,  n_classes_=2,  n_estimators=200,  n_features_=161,  n_jobs=-1,  n_outputs_=1,  oob_score=False,  random_state=5609,  warm_start=False |
| Gradient Boosting Classifier | alpha=0.9,  ccp_alpha=0.0,  criterion='friedman_mse',  learning_rate= 0.004681258334091889,  loss='deviance',  max_depth=11,  max_features= 0.857489038047461,  max_features_= 138,  max_leaf_nodes=None,  min_impurity_decrease= 0.001301812539371827,  min_impurity_split=None,  min_samples_leaf=1,  min_samples_split=7,  min_weight_fraction_leaf=0.0,  n_classes_=2,  n_estimators=279,  n_features_=161,  n_iter_no_change=None,  presort='deprecated',  random_state=5609,  subsample= 0.2822507076801092,  tol=0.0001,  validation_fraction=0.1,  warm_start=False |
| CatBoost Classifier | best_iteration_=None,  learning_rate= 0.009305999614298344,  n_features_in_=0,  random_seed_=5609,  tree_count_=1000 |
| XGBoost Classifier | best_iteration=34,  best_ntree_limit=35,  booster= 'gbtree',  colsample_bylevel=1,  colsample_bynode=1,  colsample_bytree= 0.554589340833863,  early_stopping_rounds=None,  enable_categorical=False,  eval_metric=None,  gamma=0,  gpu_id=1,  grow_policy='depthwise',  importance_type=None,  interaction_constraints='',  learning_rate= 0.16462165764195888,  max_bin=256,  max_cat_to_onehot=4,  max_delta_step=0,  max_depth=11,  max_leaves=0,  min_child_weight=3,  missing=nan,  monotone_constraints='()',  n_classes_=2,  n_estimators=35,  n_features_in_=161,  n_jobs=-1,  num_parallel_tree=1,  objective='binary:logistic',  predictor='auto',  random_state=5609,  reg_alpha= 1.4383342779621466,  reg_lambda= 0.24909155068537095,  sampling_method='uniform',  scale_pos_weight= 5.8185375030468585,  subsample= 0.48732321279930685,  tree_method='auto',  use_label_encoder=False,  validate_parameters=1 |
| Light Gradient Boosting Classifier | bagging_fraction=0.8712641312522402,  bagging_freq=4,  best_iteration_=None,  boosting_type='gbdt',  class_weight=None,  colsample_bytree=1.0,  evals_result_=None,  feature_fraction=0.5692523918483772,  fitted_=True,  importance_type='split',  learning_rate= 0.08948939260554069,  max_depth=-1,  min_child_samples=52,  min_child_weight=0.001,  min_split_gain=0.2756903122220554,  n_classes_=2,  n_estimators=73,  n_features_=161,  n_jobs=-1,  num_leaves=217,  objective=None,  objective_='binary',  random_state=5609,  reg_alpha=8.50276925410656e-08,  reg_lambda=0.2916940533037764,  silent='warn',  subsample=1.0,  subsample_for_bin=200000,  subsample_freq=0 |
| Random Forest Classifier | bootstrap=False,  ccp_alpha=0.0,  class_weight={},  criterion='entropy',  max_depth=5,  max_features=0.5148562351753774,  max_leaf_nodes=None,  max_samples=None,  min_impurity_decrease=2.4223493973000003e-07,  min_impurity_split=None,  min_samples_leaf=5,  min_samples_split=10,  min_weight_fraction_leaf=0.0,  n_classes_=2,  n_estimators=38,  n_features_=161,  n_jobs=-1,  n_outputs_=1,  oob_score=False,  random_state=5609,  warm_start=False |
| Linear Discriminant Analysis | n_components=None,  n_features_in_=161,  priors=None,  shrinkage= None,  solver= 'svd',  store_covariance=False,  tol=0.0001 |
