# Supplementary material for: ENCAP: Computational prediction of tumor T cell antigens with ensemble classifiers and diverse sequence features
Source: PLoS One. 2024 Jul 18;19(7):e0307176. doi: 10.1371/journal.pone.0307176 (PMC11257298; doi:10.1371/journal.pone.0307176)
Supplement: S3 Table — (DOCX) [file pone.0307176.s007.docx]

**S3 Table.** Hyperparameters of ML models trained with DS2-CV

| Model | Parameters |
| --- | --- |
| Extra Trees Classifier | bootstrap=False,  ccp_alpha=0.0,  class_weight=None,  criterion= 'gini'  max_depth=None,  max_features=auto,  max_leaf_nodes=None,  max_samples=None,  min_impurity_decrease=0.0,  min_impurity_split=None,  min_samples_leaf=1,  min_samples_split=2  min_weight_fraction_leaf=0.0,  n_classes_=2,  n_estimators=100,  n_features_=218,  n_jobs=-1,  n_outputs_=1,  oob_score=False,  random_state=5609 |
| Gradient Boosting Classifier | alpha=0.9,  ccp_alpha=0.0,  criterion='friedman_mse',  learning_rate= 0.018578097694156127,  loss='deviance',  max_depth=5,  max_features=0.4257086082004754,  max_features_=92,  max_leaf_nodes=None,  min_impurity_decrease=0.11150654633630529,  min_impurity_split=None,  min_samples_leaf=5,  min_samples_split=7,  min_weight_fraction_leaf=0.0,  n_classes_=2,  n_estimators=202,  n_features_=218,  n_iter_no_change=None,  presort='deprecated',  random_state=5609,  subsample=0.8124356660648147,  tol=0.0001,  validation_fraction=0.1  warm_start=False |
| CatBoost Classifier | best_iteration_=None  learning_rate= 0.01006999984383583,  n_features_in_=0,  random_seed_=5609,  tree_count_=1000 |
| XGBoost Classifier | best_iteration=99,  best_ntree_limit=100,  booster= 'gbtree'  colsample_bylevel=1,  colsample_bynode=1,  colsample_bytree=1,  early_stopping_rounds=None,  enable_categorical=False,  eval_metric=None,  gamma=0,  gpu_id=1,  grow_policy='depthwise',  importance_type=None,  interaction_constraints='',  learning_rate= 0.300000012,  max_bin=256,  max_cat_to_onehot=4,  max_delta_step=0,  max_depth=6,  max_leaves=0,  min_child_weight=1,  missing=nan,  monotone_constraints='()',  n_classes_=2,  n_estimators=100,  n_features_in_=218,  n_jobs=-1,  num_parallel_tree=1,  objective='binary:logistic',  predictor='auto',  random_state=5609,  reg_alpha=0,  reg_lambda=1,  sampling_method='uniform',  scale_pos_weight=1,  subsample=1,  tree_method='auto',  use_label_encoder=False,  validate_parameters=1 |
| Light Gradient Boosting Classifier | bagging_fraction=0.8536409324548491,  bagging_freq=5,  best_iteration_=None,  boosting_type='gbdt',  class_weight=None,  colsample_bytree=1.0,  evals_result_=None,  feature_fraction=0.4552730556035711,  fitted_=True,  importance_type='split',  learning_rate= 0.4702740978981127,  max_depth=-1,  min_child_samples=29,  min_child_weight=0.001,  min_split_gain=0.2456987470943368,  n_classes_=2,  n_estimators=93,  n_features_=218,  n_jobs=-1,  num_leaves=98,  objective=None,  objective_='binary',  random_state=5609,  reg_alpha=3.1683904765192795e-09,  reg_lambda=0.00010627784655255073,  silent='warn',  subsample=1.0,  subsample_for_bin=200000,  subsample_freq=0 |
| Random Forest Classifier | bootstrap=True,  ccp_alpha=0.0,  class_weight='balanced',  criterion='entropy',  max_depth=7,  max_features=0.6999924792767795,  max_leaf_nodes=None,  max_samples=None,  min_impurity_decrease=1.3372772636514997e-08,  min_impurity_split=None,  min_samples_leaf=2,  min_samples_split=6,  min_weight_fraction_leaf=0.0,  n_classes_=2,  n_estimators=163,  n_features_=218,  n_jobs=-1,  n_outputs_=1,  oob_score=False,  random_state=5609,  warm_start=False |
| Linear Discriminant Analysis | n_components=None,  n_features_in_=218,  priors=None,  shrinkage= 0.4467162906631396,  solver= 'eigen',  store_covariance=False,  tol=0.0001 |
