# Supplementary material for: ENCAP: Computational prediction of tumor T cell antigens with ensemble classifiers and diverse sequence features
Source: PLoS One. 2024 Jul 18;19(7):e0307176. doi: 10.1371/journal.pone.0307176 (PMC11257298; doi:10.1371/journal.pone.0307176)
Supplement: S4 Table — (DOCX) [file pone.0307176.s008.docx]

**S4 Table.** List of 161 selected feature subset for DS1 (sorted in alphabetic order).

| ABHPRK__56  Aliphatic_Index  Boman_Index  Calculate_mw  Cougar__21  Cougar__22  Cougar__23  Cougar__24  Cougar__25  Cougar__6  CTDD_hydrophobicity_PRAM900101.1.residue100  CTDD_hydrophobicity_PRAM900101.1.residue75  CTDD_normwaalsvolume.3.residue100  CTDD_polarizability.3.residue100  DDE_AC  DDE_AH  DDE_AM  DDE_AR  DDE_CA  DDE_CC  DDE_CF  DDE_CI  DDE_CK  DDE_CM  DDE_CN  DDE_CQ  DDE_CR  DDE_CV  DDE_CW  DDE_CY  DDE_DA  DDE_DC  DDE_DH  DDE_DQ  DDE_EH  DDE_EL  DDE_EQ  DDE_EW  DDE_FA  DDE_FI  DDE_FQ  DDE_FW  DDE_HA  DDE_HC  DDE_HG | DDE_HH  DDE_HI  DDE_HN  DDE_HR  DDE_ID  DDE_IH  DDE_IM  DDE_IT  DDE_LM  DDE_MC  DDE_ME  DDE_MF  DDE_MH  DDE_MK  DDE_MM  DDE_MN  DDE_MP  DDE_MW  DDE_MY  DDE_NA  DDE_NM  DDE_NP  DDE_NW  DDE_PC  DDE_PF  DDE_PM  DDE_PW  DDE_PY  DDE_QC  DDE_QG  DDE_QI  DDE_QW  DDE_RQ  DDE_SM  DDE_TH  DDE_TI  DDE_TM  DDE_TP  DDE_TQ  DDE_VW  DDE_VY  DDE_WA  DDE_WC  DDE_WD  DDE_WE | DDE_WF  DDE_WH  DDE_WI  DDE_WK  DDE_WL  DDE_WS  DDE_WV  DDE_WW  DDE_YA  DDE_YE  DDE_YG  DDE_YI  DDE_YL  DDE_YQ  DDE_YW  Ez__1  Ez__10  Ez__11  Ez__12  Ez__14  Ez__15  Ez__7  Ez__8  Ez__9  formula_H  Hopp_woods  Length  MotifBitVec_FATP  MotifBitVec_IPRHL  MotifBitVec_LLD  MotifBitVec_LMK  MotifBitVec_RLAE  MotifBitVec_SLADTN  MotifBitVec_VFGI  MSW__10  MSW__11  MSW__12  MSW__13  MSW__14  MSW__17  MSW__18  MSW__19  MSW__2  MSW__20  MSW__21 | MSW__22  MSW__3  MSW__4  MSW__5  MSW__6  MSW__7  MSW__8  MSW__9  OVPC_Aliphatic  QSO3_G1  QSO3_G2  QSO3_G3  QSO3_SC_L  QSO3_SC_S  QSO3_SC1  QSO3_SC2  QSO3_SC3  Z3__6  Z5__31  Z5__6  Z5__61  Z5__64 |
| --- | --- | --- | --- |
