# Supplementary material for: ENCAP: Computational prediction of tumor T cell antigens with ensemble classifiers and diverse sequence features
Source: PLoS One. 2024 Jul 18;19(7):e0307176. doi: 10.1371/journal.pone.0307176 (PMC11257298; doi:10.1371/journal.pone.0307176)
Supplement: S5 Table — (DOCX) [file pone.0307176.s009.docx]

**S5 Table.** List of 218 selected feature subset for DS2 (sorted in alphabetic order)

| AAC_C  AAC_L  ABHPRK__1  ABHPRK__16  ABHPRK__17  ABHPRK__20  ABHPRK__76  ABHPRK__77  APAAC_Pc1.C  APAAC_Pc1.I  APAAC_Pc1.K  APAAC_Pc1.L  APAAC_Pc1.R  Boman_Index  Calculate_charge  Charge_acid  Cougar__11  Cougar__12  Cougar__13  Cougar__14  Cougar__15  Cougar__16  Cougar__2  Cougar__21  Cougar__22  Cougar__24  Cougar__26  Cougar__27  Cougar__28  Cougar__29  Cougar__3  Cougar__30  Cougar__6  Cougar__7  CTDC_charge.G2  CTDC_charge.G3  CTDC_hydrophobicity_ENGD860101.G1  CTDC_hydrophobicity_ENGD860101.G3  CTDC_hydrophobicity_FASG890101.G1  CTDC_hydrophobicity_PONP930101.G1  CTDC_hydrophobicity_PONP930101.G3  CTDC_hydrophobicity_PRAM900101.G1  CTDC_hydrophobicity_PRAM900101.G3  CTDC_polarity.G1  CTDC_polarity.G3  CTDC_solventaccess.G2  CTDD_charge.1.residue0  CTDD_charge.1.residue100  CTDD_charge.1.residue25  CTDD_charge.1.residue50  CTDD_charge.1.residue75  CTDD_charge.2.residue0  CTDD_charge.2.residue25  CTDD_charge.3.residue75 | CTDD_hydrophobicity_ARGP820101.1.residue0  CTDD_hydrophobicity_ARGP820101.1.residue25  CTDD_hydrophobicity_ARGP820101.3.residue0  CTDD_hydrophobicity_ARGP820101.3.residue100  CTDD_hydrophobicity_ARGP820101.3.residue25  CTDD_hydrophobicity_CASG920101.1.residue0  CTDD_hydrophobicity_CASG920101.1.residue25  CTDD_hydrophobicity_CASG920101.2.residue0  CTDD_hydrophobicity_CASG920101.2.residue25  CTDD_hydrophobicity_CASG920101.2.residue50  CTDD_hydrophobicity_ENGD860101.1.residue0  CTDD_hydrophobicity_ENGD860101.1.residue100  CTDD_hydrophobicity_ENGD860101.1.residue25  CTDD_hydrophobicity_ENGD860101.1.residue50  CTDD_hydrophobicity_ENGD860101.3.residue100  CTDD_hydrophobicity_FASG890101.1.residue0  CTDD_hydrophobicity_FASG890101.1.residue25  CTDD_hydrophobicity_FASG890101.1.residue50  CTDD_hydrophobicity_FASG890101.3.residue0  CTDD_hydrophobicity_FASG890101.3.residue25  CTDD_hydrophobicity_PONP930101.1.residue0  CTDD_hydrophobicity_PONP930101.1.residue25  CTDD_hydrophobicity_PONP930101.2.residue0  CTDD_hydrophobicity_PONP930101.2.residue25  CTDD_hydrophobicity_PONP930101.3.residue0  CTDD_hydrophobicity_PONP930101.3.residue25  CTDD_hydrophobicity_PRAM900101.1.residue0  CTDD_hydrophobicity_PRAM900101.1.residue25  CTDD_hydrophobicity_PRAM900101.1.residue50  CTDD_hydrophobicity_PRAM900101.1.residue75  CTDD_hydrophobicity_PRAM900101.2.residue100  CTDD_hydrophobicity_PRAM900101.3.residue0  CTDD_hydrophobicity_PRAM900101.3.residue100  CTDD_hydrophobicity_PRAM900101.3.residue25  CTDD_hydrophobicity_ZIMJ680101.2.residue100  CTDD_hydrophobicity_ZIMJ680101.3.residue0  CTDD_hydrophobicity_ZIMJ680101.3.residue100  CTDD_hydrophobicity_ZIMJ680101.3.residue25  CTDD_normwaalsvolume.2.residue0  CTDD_normwaalsvolume.2.residue100  CTDD_normwaalsvolume.2.residue25  CTDD_normwaalsvolume.3.residue0  CTDD_normwaalsvolume.3.residue100  CTDD_normwaalsvolume.3.residue25  CTDD_normwaalsvolume.3.residue50  CTDD_polarity.1.residue0  CTDD_polarity.1.residue25  CTDD_polarity.2.residue25  CTDD_polarity.3.residue0  CTDD_polarity.3.residue25  CTDD_polarity.3.residue50  CTDD_polarity.3.residue75  CTDD_polarizability.2.residue0  CTDD_polarizability.2.residue100  CTDD_polarizability.2.residue25 | CTDD_polarizability.3.residue0  CTDD_polarizability.3.residue100  CTDD_polarizability.3.residue25  CTDD_polarizability.3.residue50  CTDD_solventaccess.1.residue0  CTDD_solventaccess.1.residue25  CTDD_solventaccess.1.residue50  CTDD_solventaccess.2.residue0  CTDD_solventaccess.2.residue25  CTDD_solventaccess.2.residue50  CTDD_solventaccess.2.residue75  CTDD_solventaccess.3.residue0  CTDD_solventaccess.3.residue100  CTDD_solventaccess.3.residue25  CTDD_solventaccess.3.residue50  DDR_C  DDR_K  Ez__1  Ez__10  Ez__11  Ez__12  Ez__13  Ez__14  Ez__15  Ez__16  Ez__17  Ez__18  Ez__20  Ez__21  Ez__22  Ez__23  Ez__24  Ez__25  Ez__27  Ez__29  Ez__6  Ez__7  Ez__8  Ez__9  formula_S  GAAC_negativecharge  GAAC_postivecharge  Geary_BIGC670101.lag1  Geary_CHAM820101.lag1  Geary_CHAM820102.lag1  Isoelectric_point  MotifBitVec_ADV  MotifBitVec_AGIGIL  MotifBitVec_AQID  MotifBitVec_AVADE  MotifBitVec_EFP  MotifBitVec_ITD  MotifBitVec_LMK  MotifBitVec_VDEV | MSW__18  MSW__20  MSW__21  MSW__23  MSW__24  MSW__26  MSW__27  MSW__6  OVP_Aliphatic_C5  OVP_Aromatic_C5  OVP_Aromatic_N2  OVP_Charged_N2  OVP_Charged_N3  OVP_Hydrophobic_N1  OVP_Hydrophobic_N2  OVP_Negative_N2  OVP_Polar_N1  OVP_Polar_N2  OVPC_Charged  OVPC_Negative  OVPC_Positive  QSO3_G1  QSO3_SC_C  QSO3_SC_L  QSO3_SC_R  QSO3_SC1  QSO3_SC2  RRI_F  SEP_C  SEP_L  SER_C  SER_L  TM_tend  Z3__12  Z3__21  Z3__26  Z3__3  Z3__6  Z5__1  Z5__16  Z5__20  Z5__21  Z5__22  Z5__31  Z5__36  Z5__41  Z5__46  Z5__51  Z5__53  Z5__56  Z5__6  Z5__61  Z5__66  Z5__71  Z5__72 |
| --- | --- | --- | --- |
