# Supplementary material for: ENCAP: Computational prediction of tumor T cell antigens with ensemble classifiers and diverse sequence features
Source: PLoS One. 2024 Jul 18;19(7):e0307176. doi: 10.1371/journal.pone.0307176 (PMC11257298; doi:10.1371/journal.pone.0307176)
Supplement: S6 Table — (DOCX) [file pone.0307176.s010.docx]

**S6 Table.** The selected numbers and sizes of feature types from the selected feature subsets of DS1 and DS2.

| DS1 | | | DS2 | | |
| --- | --- | --- | --- | --- | --- |
| Feature Type | Selected Num. | Size. | Feature Type | Selected Num. | Size |
| DDE | 91 | 400 | CTDD**^*^** | 78 | 195 |
| MSW^*^ | 19 | 30 | Ez**^*^** | 22 | 30 |
| Ez^*^ | 9 | 30 | Cougar**^*^** | 18 | 30 |
| QSO^*^ | 8 | 46 | Z5**^*^** | 17 | 75 |
| Cougar^*^ | 6 | 30 | CTDC | 12 | 39 |
| CTDD^*^ | 4 | 195 | OVP | 10 | 100 |
| Z5^*^ | 4 | 75 | MSW**^*^** | 8 | 30 |
| ABHPRK^*^ | 1 | 105 | ABHPRK**^*^** | 6 | 105 |
| Aliphatic_Index | 1 | 1 | QSO**^*^** | 6 | 46 |
| Boman_Index | 1 | 1 | APAAC | 5 | 26 |
| Calculate_mw | 1 | 1 | Z3**^*^** | 5 | 30 |
| Formula_H | 1 | 5 | Geary | 3 | 24 |
| Hopp_wood | 1 | 1 | OVPC | 3 | 10 |
| Length | 1 | 1 | AAC | 2 | 51 |
| OVPC_Aliphatic | 1 | 110 | DDR | 2 | 20 |
| Z3^*^ | 1 | 30 | GAAC | 2 | 5 |
|  |  |  | SEP | 2 | 20 |
|  |  |  | SER | 2 | 20 |
|  |  |  | Boman | 1 | 1 |
|  |  |  | Calculate_charge | 1 | 1 |
|  |  |  | charge_acid | 1 | 1 |
|  |  |  | formula_S | 1 | 1 |
|  |  |  | Isoelectric_point | 1 | 1 |
|  |  |  | RRI | 1 | 20 |
|  |  |  | TM_tend | 1 | 1 |

^*^common feature types between DS1 and DS2
