# Supplementary material for: ENCAP: Computational prediction of tumor T cell antigens with ensemble classifiers and diverse sequence features
Source: PLoS One. 2024 Jul 18;19(7):e0307176. doi: 10.1371/journal.pone.0307176 (PMC11257298; doi:10.1371/journal.pone.0307176)
Supplement: S1 Text — (DOCX) [file pone.0307176.s011.docx]

**S1 Text.** Pseudocode of ENCAP

**Algorithm ENCAP**

**Input:** CV dataset, independent test dataset

**Output:** Optimized prediction model, CV results, independent test results

**Stage 1**: Feature Engineering

1. Convert sequences from CV dataset to numerical features

2. Normalize data

3. Rank features

4. Determine the best feature number *N* using the feature subset selection algorithm

5. Obtain *M* motif-based features using a motif searching algorithm

6. Combine *N* selected features with *M* motif-based features to form the best feature subset

**Stage 2**: Hyperparameter Optimization

8. For each ML model in [CB, GBC, ET, XGB, LGBM, RF, LDA]:

a. Optimize hyperparameters of each ML model using Bayesian optimization on the CV dataset with the best feature subset

b. Determine the best hyperparameters

9. Save the optimized models

10. Perform 10-fold CV and independent test using the optimized models

11. Benchmark results with various evaluation measures

**End Algorithm**
