# Supplementary material for: ENCAP: Computational prediction of tumor T cell antigens with ensemble classifiers and diverse sequence features
Source: PLoS One. 2024 Jul 18;19(7):e0307176. doi: 10.1371/journal.pone.0307176 (PMC11257298; doi:10.1371/journal.pone.0307176)
Supplement: S2 Text — (DOCX) [file pone.0307176.s012.docx]

**S2 Text.** Pseudocode of the feature subset selection algorithm

**Algorithm Feature_Subset_Selection**

**Input:** Datasets DS1-CV and DS2-CV

**Output:** Best Feature Subsets (*BFS*) for DS1-CV and DS2-CV

1. Apply the Boruta algorithm to a given dataset (DS1-CV or DS2-CV) for generating a ranked feature list.

2. Express the ranked features as an ordered set *F* = {*f_1_*, *f_2_*, … *f_4335_*}, sorted by feature importance.

3. Define the feature subset *S_N_* = {*f_1_*, *f_2_*, … *f_N_*} where *N* is the number of top features to consider.

4. For N ranging from 50 to 410 in increments of 20:

a. Perform iterative five-fold cross-validation using *S_N_* with six ensemble predictors: RF, ET, GBC, LGBM, XGB, and CB.

b. Evaluate the results with the Matthews Correlation Coefficient (MCC).

c. Record the best MCC for each *N* as *Best_MCC_N*.

5. Determine the best feature subset *BFS* as the *S_N_* that yields the highest *Best_MCC_N*.

**End Algorithm**
